# Effectiveness of Surgery for Lumbar Spinal Stenosis: A Systematic Review and Meta-Analysis

**DOI:** 10.1371/journal.pone.0122800

**Published:** 2015-03-30

**Authors:** Gustavo C. Machado, Paulo H. Ferreira, Ian A. Harris, Marina B. Pinheiro, Bart W. Koes, Maurits van Tulder, Magdalena Rzewuska, Chris G. Maher, Manuela L. Ferreira

**Affiliations:** 1 The George Institute for Global Health, Sydney Medical School, University of Sydney, Sydney, NSW, Australia; 2 Faculty of Health Sciences, University of Sydney, Sydney, NSW, Australia; 3 South Western Sydney Clinical School, Ingham Institute for Applied Medical Research, University of New South Wales, Sydney, NSW, Australia; 4 Department of General Practice, Erasmus Medical Centre, Rotterdam, The Netherlands; 5 Department of Health Sciences, VU University, Amsterdam, The Netherlands; 6 Institute of Bone and Joint Research, Sydney Medical School, University of Sydney, Sydney, NSW, Australia; Toronto Western Hospital, CANADA

## Abstract

**Background:**

The management of spinal stenosis by surgery has increased rapidly in the past two decades, however, there is still controversy regarding the efficacy of surgery for this condition. Our aim was to investigate the efficacy and comparative effectiveness of surgery in the management of patients with lumbar spinal stenosis.

**Methods:**

Electronic searches were performed on MEDLINE, EMBASE, AMED, CINAHL, Web of Science, LILACS and Cochrane Library from inception to November 2014. Hand searches were conducted on included articles and relevant reviews. We included randomised controlled trials evaluating surgery compared to no treatment, placebo/sham, or to another surgical technique in patients with lumbar spinal stenosis. Primary outcome measures were pain, disability, recovery and quality of life. The PEDro scale was used for risk of bias assessment. Data were pooled with a random-effects model, and the GRADE approach was used to summarise conclusions.

**Results:**

Nineteen published reports (17 trials) were included. No trials were identified comparing surgery to no treatment or placebo/sham. Pooling revealed that decompression plus fusion is not superior to decompression alone for pain (mean difference –3.7, 95% confidence interval –15.6 to 8.1), disability (mean difference 9.8, 95% confidence interval –9.4 to 28.9), or walking ability (risk ratio 0.9, 95% confidence interval 0.4 to 1.9). Interspinous process spacer devices are slightly more effective than decompression plus fusion for disability (mean difference 5.7, 95% confidence interval 1.3 to 10.0), but they resulted in significantly higher reoperation rates when compared to decompression alone (28% *v* 7%, P < 0.001). There are no differences in the effectiveness between other surgical techniques for our main outcomes.

**Conclusions:**

The relative efficacy of various surgical options for treatment of spinal stenosis remains uncertain. Decompression plus fusion is not more effective than decompression alone. Interspinous process spacer devices result in higher reoperation rates than bony decompression.

## Introduction

Lumbar spinal stenosis is a narrowing of the spinal canal by surrounding bone and soft tissues that compromises neural structures. Radiographic findings of spinal stenosis are highly prevalent [1], and 85% of patients typically present with significant long-term symptoms of intermittent neurogenic claudication (radicular pain during walking or standing that resolves with lumbar flexion) [2]. When refractory to conservative treatment, patients are commonly referred for surgery [3, 4]. As a result, the number of surgical procedures performed for lumbar spinal stenosis has increased steadily over the years (e.g., the rates of complex fusion surgery had a 15-fold increase between 2002 and 2007) [5], with costs reaching USD $1.65 billion per year [6]. However, there is still a substantial variation in the surgical technique chosen by surgeons [7, 8], although no clear superiority of one technique over the others has been yet identified [9–11].

The current evidence suggests that surgery for spinal stenosis is more effective than conservative treatment when the latter has failed for up to six months [12, 13]. For instance, in the Spine Patient Outcomes Research Trial (SPORT) patients treated surgically reported lower pain levels compared to patients assigned to nonsurgical care [14]. The gold standard surgical approach for lumbar spinal stenosis is bony decompression by laminectomy [15, 16]. However, due to the occurrence of complications associated with this technique [17], less invasive surgical techniques have been proposed, such as unilateral or bilateral laminotomies [18–20], and spinous process split–laminectomy [21]. Additionally, as spinal instability is a frequent finding following bony decompression [22, 23], surgical fusion has been recommended in addition to decompression of the spinal canal for the management of some patients with spinal stenosis [24]. However, this practice can be associated with higher reoperation rates, post-surgical complications, and costs when compared to decompression alone [25]. Although many surgical techniques are available for the management of lumbar spinal stenosis, there seems to be a paucity of evidence supporting this rapid evolution of surgical techniques, and clinicians are usually asked to rely on their own opinions and experiences [26].

Therefore, in this systematic review we aimed to determine the efficacy of surgery in the management of patients with lumbar spinal stenosis and the comparative effectiveness between commonly performed surgical techniques to treat this condition.

## Material and Methods

### Data sources and search

We conducted a systematic review and meta-analysis following the recommendations of the PRISMA statement [27]. The methods of this review have been previously registered with PROSPERO, number CRD42013005901. We performed a systematic electronic search on MEDLINE, EMBASE, AMED, CINAHL, Web of Science, LILACS and Cochrane Central Register of Controlled Trials from the date of inception until June 2014. The search strategy is in S1 Table. Hand searches of references were also conducted on relevant reviews and included studies.

### Study selection

Two independent reviewers (GM and MP/MR) performed the selection of studies and consensus was used to resolve any disagreement. To be included, studies needed to be full published randomised controlled trials comparing the efficacy of surgery to no treatment, placebo/sham, or comparing the effectiveness of different types of surgical procedures. Trials were included if they explicitly reported that subjects were treated for lumbar spinal stenosis, despite its anatomical classification (central, foraminal or lateral), or diagnostic criteria. There were no restrictions regarding intensity or duration of symptoms, language or publication date. Studies of patients with trauma, tumour, and previous spine surgery were excluded. As degenerative spondylolisthesis is a common finding in patients with lumbar spinal stenosis, only trials including patients with spondylolisthesis greater than grade I were excluded. Review articles, guidelines, observational studies, trials comparing different types of fusion techniques, and surgery for cervical spine stenosis were also excluded.

### Data extraction and quality assessment

Using a standardised extraction form, data from each included study were independently extracted by two reviewers (GM and MP) and consensus used to resolve any disagreement. The following information from each study was extracted: participants’ characteristics (age, stenosis duration and diagnosis criteria), type of surgery and outcome measure. Primary outcomes of interest were pain (e.g., back pain, leg pain, overall pain), disability (e.g., Oswestry Disability Index, walking ability), quality of life, and recovery. Quality of life measures of our interest included for example total scores of the 36-item short-form health survey (SF-36) or from the EuroQol questionnaire. However, none of the trials included in our review reported the total scores of these measures. Instead, they reported scores for the sub-items (e.g., Physical Function or Physical Component Scores) and therefore could not be included in our analyses. Recovery was measured using the differences between preoperative and postoperative Japanese Orthopaedic Association (JOA) scores and reported in the included trials. Secondary outcomes included perioperative surgical data (e.g., blood loss, operation time, length of hospitalisation), complications, reoperations, and costs. To enable cross-trial comparisons, terms used to describe surgical complications were coded based on previously established standard definitions for common complications post spine surgery [28]. We extracted sample sizes, means (final values) and standard deviations for continuous outcomes, and number of cases for dichotomous outcomes. If trials reported incomplete data, authors were contacted for further information. If authors were unavailable, missing data were imputed according to recommendations in the Cochrane Handbook for Systematic Reviews of Interventions [29].

We used the Physiotherapy Evidence Database (PEDro) scale to assess the methodological quality of the included studies. The PEDro scale is widely used to assess the quality of clinical trials in various areas of medicine [30], and consists of an 11-item checklist that has been shown to be a valid and reliable tool [31, 32]. Two raters (GM and MR) independently assessed the methodological quality of each included study and a third author resolved any disagreement. Trials were considered to be of high methodological quality when the PEDro final score was ≥6 points.

### Data synthesis and analysis

All data on leg pain, back pain or overall pain were extracted from included trials. If trials reported more than one measure of pain intensity (e.g., back and leg pain), the more severe measure at baseline was included in the analyses. Pain and disability outcome measures were converted to scales from 0 (no pain or disability) to 100 (worst possible pain or disability). For data synthesis, follow-up times were categorized as short-term (less than 12 months) and long-term (12 months or more). If studies reported multiple time points within each category, the time point closest to three months for the short-term, and 12 months for the long-term were used. When more than one scale to measure pain or disability was reported, the one cited by the authors as the primary outcome was used. When studies reported results for more than two intervention groups, we combined similar groups according to the recommendations in the Cochrane Handbook [29].

Trials were grouped according to type of surgery comparison, outcomes, and assessment time points. We used a random-effects model to calculate mean differences (MD) and 95% confidence intervals (CI) for continuous measures. For dichotomous outcomes, risk ratio (RR) and 95% CI was used. Descriptive and inferential statistics were used to present complication and reoperation rates, with a significance level at 5%. The I^2^ statistic was used to assess heterogeneity between trials, and values higher than 50% were defined to identify high heterogeneity [33]. Comprehensive Meta-Analysis version 2.2.064 (Englewood, NJ, USA, 2011) was used for all analyses.

### Grading the evidence and applicability

The GRADE (Grading of Recommendations Assessment, Development and Evaluation) system was used to assess the overall quality of the evidence and strength of recommendations for each outcome measure [34]. The quality of evidence was downgraded by one level according to the following criteria: limitation of study design (> 25% of the studies with low methodological quality [PEDro score < 6]), inconsistency of results (statistically significant heterogeneity [I^2^ > 50%] or ≤ 75% of trials with findings in the same direction), and imprecision (wide confidence intervals or total number of participants < 300 for each pooled analysis). The indirectness criterion was not considered in this review because we included a specific population with relevant outcomes and direct comparisons. Where only single trials were available, evidence from studies with < 300 participants was downgraded for inconsistency and imprecision and rated as “low quality” evidence. They could be further downgraded to “very low quality” evidence if limitations of study design were found. The quality of evidence was defined as: “high quality”, “moderate quality”, “low quality”, and “very low quality” [34].

## Results

### Study characteristics

**Fig 1:**
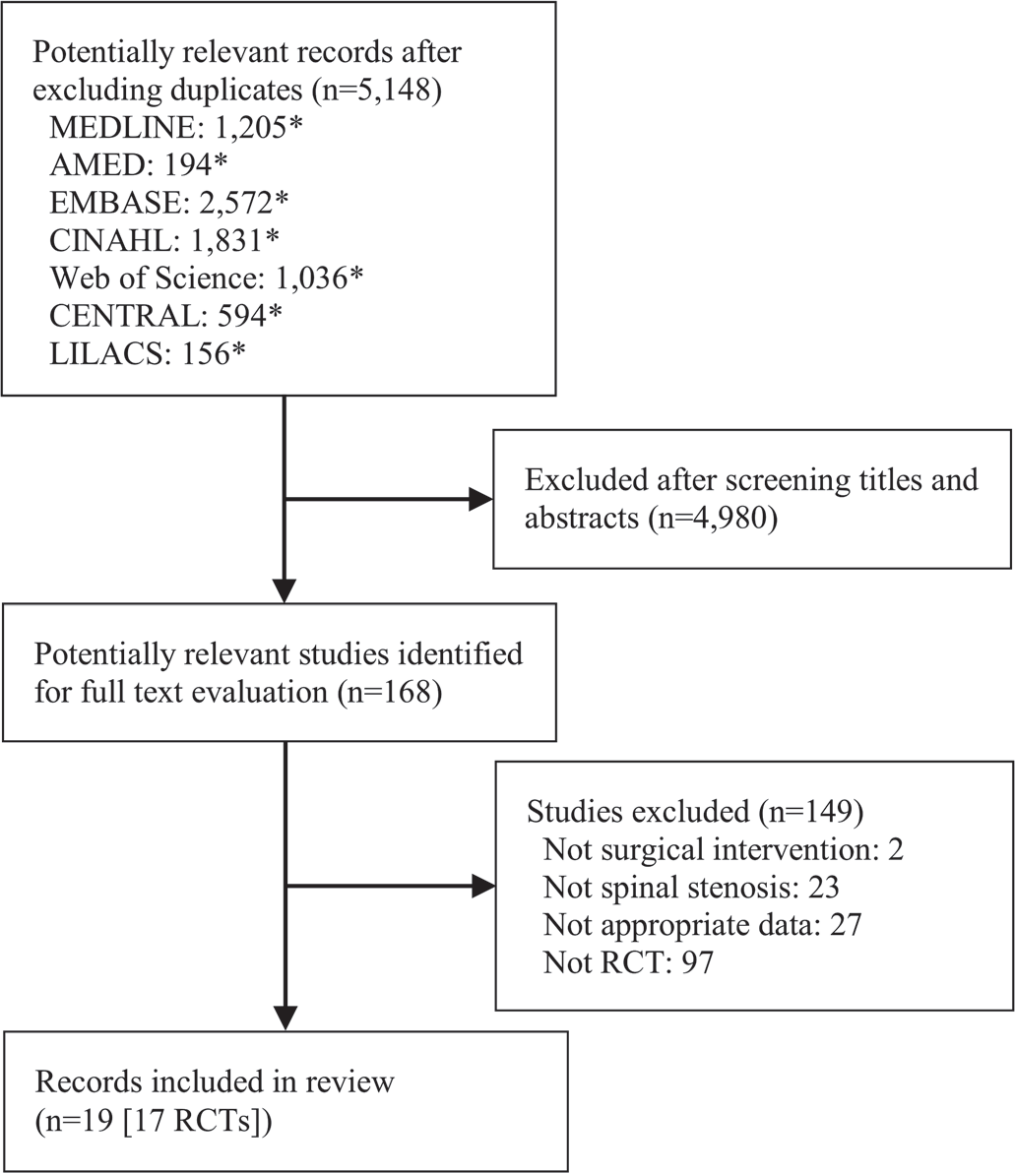
Flow Diagram of Studies Included in the Systematic Review. RCT = randomised controlled trial. *Number of citations includes duplicates.

A total of 7,284 records were identified. After excluding duplicates 5,148 titles and abstracts were reviewed, and 168 full text records were assessed. Of these, 19 published reports (17 randomised controlled trials) remained eligible for inclusion in our review [9–11, 35–50]. Flow chart diagram of included studies with the main reasons for exclusion are shown in Fig 1. Two records reported results from the same trial, a subgroup analysis and overall results [48, 49]. Therefore, only the full report was included in our analysis. One trial was published in English as well as in German [9, 50], and as they reported similar results we included the English publication in our analyses. All remaining trials included in this review were published in English and therefore no translation was required.

### Participant characteristics

The 17 included trials investigated a total of 1,554 patients and most studies defined lumbar spinal stenosis based on clinical assessment with a concordant imaging diagnosis [9–11, 36–38, 40–47, 49]. One study included patients based solely on imaging diagnosis [35], and another study used clinical assessment only [39]. Fourteen out of 17 trials (82%) explicitly reported including only patients who had failed to improve with conservative treatment [9–11, 36–38, 40–43, 45–47, 49]. The characteristics of included studies and participants are described in Table 1.

**Table 1:** Characteristics of Included Studies.

**Study**	**Details of Participants**	**Surgery Type**	**Outcomes (Time Point)**
**Decompression *v* Decompression+Fusion**			
Bridwell et al, 1993	44 patients (G1 = 10, G2 and G3 combined = 34); Mean age (range): 66.1 (46–79) years; Stenosis duration: NR	G1: Decompression; G2: Decompression plus fusion; G3: As G2 with instrumentation	Walking ability, complications, reoperations; at mean follow-up of 3.1 years
Grob et al, 1995	45 patients (G1 = 15, G2 and G3 combined = 30); Mean age (range): 67 (48–87) years; Stenosis duration: NR	G1: Decompression; G2: Decompression plus fusion (most stenotic segment); G3: As G2 (all stenotic segments)	VAS (overall pain), walking ability, operation time, blood loss, complications, reoperations; at 24 months
Hallet et al, 2007	44 patients (G1 = 14, G2 and G3 combined = 30); Mean age (range): 57 (34–75) years; Stenosis duration: NR	G1: Decompression; G2: Decompression plus instrumented postero-lateral fusion; G3: As G2 plus transforaminal interbody fusion	VAS (back pain), RMDQ, operation time, blood loss, reoperations, costs; at 24 months
**Laminectomy *v* Laminotomy**			
Postacchini et al, 1992	67 patients (G1 = 35, G2 = 32); Mean age (range): 57 (43–79) years; Stenosis duration: NR	G1: Multiple laminotomy; G2: Laminectomy	VAS (leg pain, radicular symptoms), operating time, blood loss, complications; at mean follow-up of 3.7 years
Thome et al, 2005	120 patients (G1 and G2 combined = 80, G3 = 40); Mean age (SD): 68 (9) years; Mean stenosis duration (SD): 20.2 (29.7) months	G1: Bilateral laminotomy; G2: Unilateral laminotomy; G3: Laminectomy	VAS (overall pain), RMDQ, walking distance, duration of operation, blood loss, complications, reoperations; at 3 and 12 months
Cavusoglu et al, 2007	100 patients (G1 = 50, G2 = 50); Mean age (SD): 69.2 (12.2) years; Stenosis duration: 8 to 60 months	G1: Unilateral laminectomy; G2: Unilateral laminotomy	SF-36 body pain, ODI, complications; at 3 months and 4 to 7 years
Celik et al, 2010	80 patients (G1 = 40, G2 = 40); Mean age (SD): G1 = 61 (13), G2 = 59 (14) years; Stenosis duration: NR	G1: Total laminectomy; G2: Bilateral microdecompressive laminotomy	VAS (leg pain), ODI, walking distance, operation time, blood loss, complications, reoperations; at 3 and 12 months
Gurelik et al, 2012	52 patients (G1 = 26, G2 = 26); Mean age (SD): G1 = 60.7 (10), G2 = 57.5 (8.5) years; Stenosis duration: NR	G1: Unilateral laminotomy; G2: Laminectomy	ODI, walking distance; at 6 months
**Laminectomy *v* Split-laminectomy/laminotomy**			
Watanabe et al, 2011	41 patients (G1 = 22, G2 = 19); Mean age (SD): G1 = 69 (10), G2 = 71 (8) years; Stenosis duration: NR	G1: Spinous process-splitting laminectomy; G2: Laminectomy	JOA, recovery, operation time, blood loss, reoperations; at 12 months
Liu et al, 2013	56 patients (G1 = 27, G2 = 29); Mean age (SD): G1 = 59.4 (4.7), G2 = 61.1 (3.1) years; Mean stenosis duration: G1 = 6.5, G2 = 5.9 years	G1: Modified unilateral laminotomy; G2: Laminectomy	VAS (leg pain), JOA, operation time, blood loss; at 24 months
Rajasekaran et al, 2013	51 patients (G1 = 28, G2 = 23); Mean age (SD): G1 = 57.3 (11.2), G2 = 54.5 (8.2) years; Stenosis duration: NR	G1: Spinous process-splitting laminectomy; G2: Laminectomy	VAS (leg pain), JOA, recovery, operation time, blood loss, hospitalisation, complications, reoperations; at 6 and 12 months
**Laminectomy/laminotomy *v* Endoscopic-laminectomy/laminotomy**			
Ruetten et al, 2009	192 patients (G1 = 100, G2 = 92); Mean age (range): 64 (38–86) years; Mean stenosis duration (range): 19 (2–78) months	G1: Laminotomy; G2: Full endoscopic laminotomy	ODI, operation time, complications, reoperations; at 3 and 12 months
Yagi et al, 2009	41 patients (G1 = 20, G2 = 21); Mean age (range): G1 = 73.3 (63–79), G2 = 70.8 (66–73) years; Stenosis duration: NR	G1: Microendoscopic laminectomy; G2: Laminectomy	JOA, operation time, blood loss, hospitalisation; at 3 and 12 months
**Laminectomy/laminotomy *v* Interspinous process spacer device**			
Stromqvist et al, 2013	100 (G1 = 50, G2 = 50); Mean age (range): 69 (49–89) years; Stenosis duration: NR	G1: Laminectomy/laminotomy; G2: X-Stop device	VAS (leg pain), ZCQ (physical function), operation time, complications, reoperations; at 6 and 12 months
Moojen et al, 2013	159 patients (G1 = 79; G2 = 80); Median age (range): G1 = 64 (47–83), G2 = 66 (45–83) years; Mean stenosis duration: G1 = 22, G2 = 23 months	G1: Laminotomy/fecetectomy; G2: Coflex Device	VAS (leg pain), ZCQ (physical function), walking ability, operation time, hospitalisation, complications, reoperations; at 6 and 12 months
**Decompression+Fusion *v* Interspinous process spacer device**			
Azzazi et al, 2010	60 patients (G1 = 30, G2 = 30); Mean age (range): 56.3 (27–79) years; Mean stenosis duration: 5.3 (0.2–36.9) years	G1: Decompression plus transpedicular screw fixation; G2: X-Stop device	VAS (leg pain), ODI, operation time, hospitalisation, complications; at 24 months
Davis et al, 2013	322 patients (G1 = 107, G2 = 215); Mean age (SD): G1 = 64.1 (9); G2 = 62.1 (9.2); Stenosis duration: NR	G1: Decompression plus transpedicular screw fixation; G2: Coflex device	VAS (leg pain), ODI, operation time, blood loss, hospital length of stay, complications, reoperations; at 24 months

SD = standard deviation; NR = not reported; VAS = visual analogue scale; RMDQ = Roland Morris Disability questionnaire; ODI = Oswestry Disability Index; SF-36 = 36-item short-form health survey; JOA = Japanese Orthopaedic Association Score; ZCQ = Zurich Claudication Questionnaire

### Quality assessment

**Fig 2:**
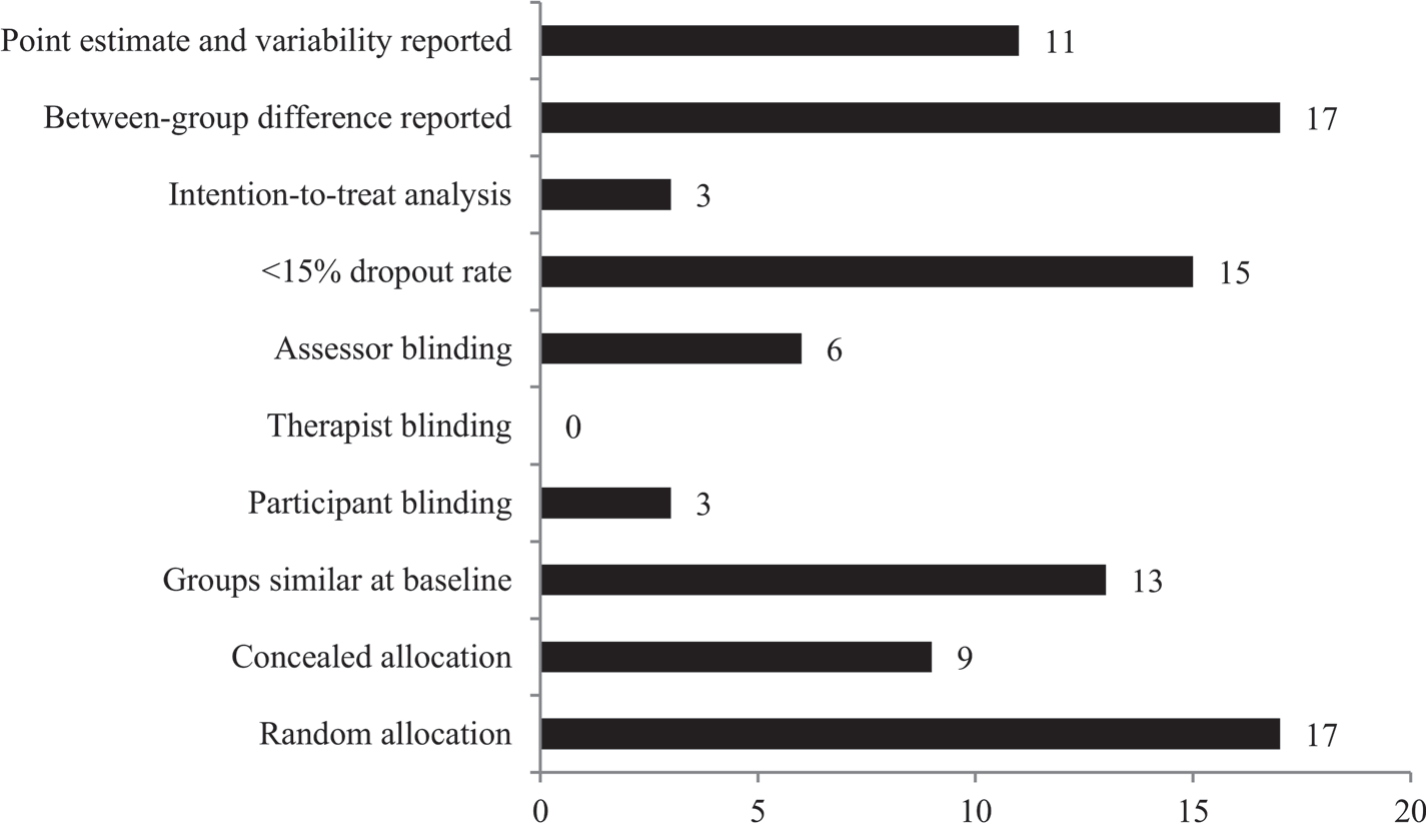
Risk of Bias (PEDro) Criteria and Number of Trials in Each Category. PEDro = Physiotherapy Evidence Database.

The methodological quality of the included trials revealed a mean score of 5.5 (standard deviation 1.8) using the PEDro scale (range, 0 to 10 score). The most common methodological flaws were lack of blinding (therapist, patient and assessor) and failure to use an intention-to-treat analysis. The three studies that blinded the patients reported that all patients gave informed consent and only one trial described that patients were informed about the operation, timing, and potential complications before the procedure [37, 46, 49]. Only half of the included trials reported concealed allocation (Fig 2). Full details of the final PEDro score for each trial is presented in S2 Table. Given the small number of trials included in each meta-analysis, small study bias analysis was not possible.

### Interventions

**Fig 3:**
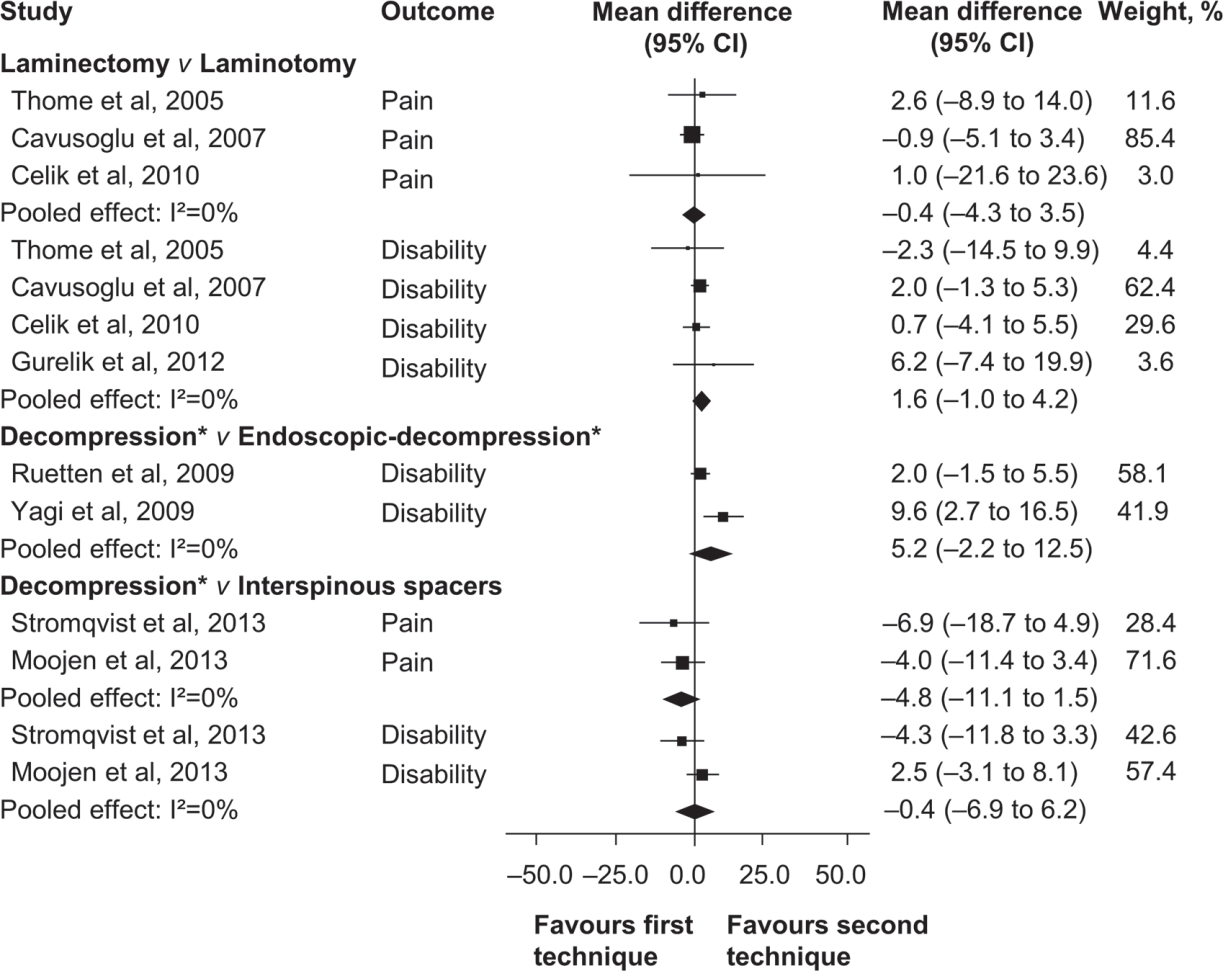
Mean Difference for Pain and Disability at Short-term Follow-up (less than 12 months). *Decompression technique is laminectomy or laminotomy.

**Fig 4:**
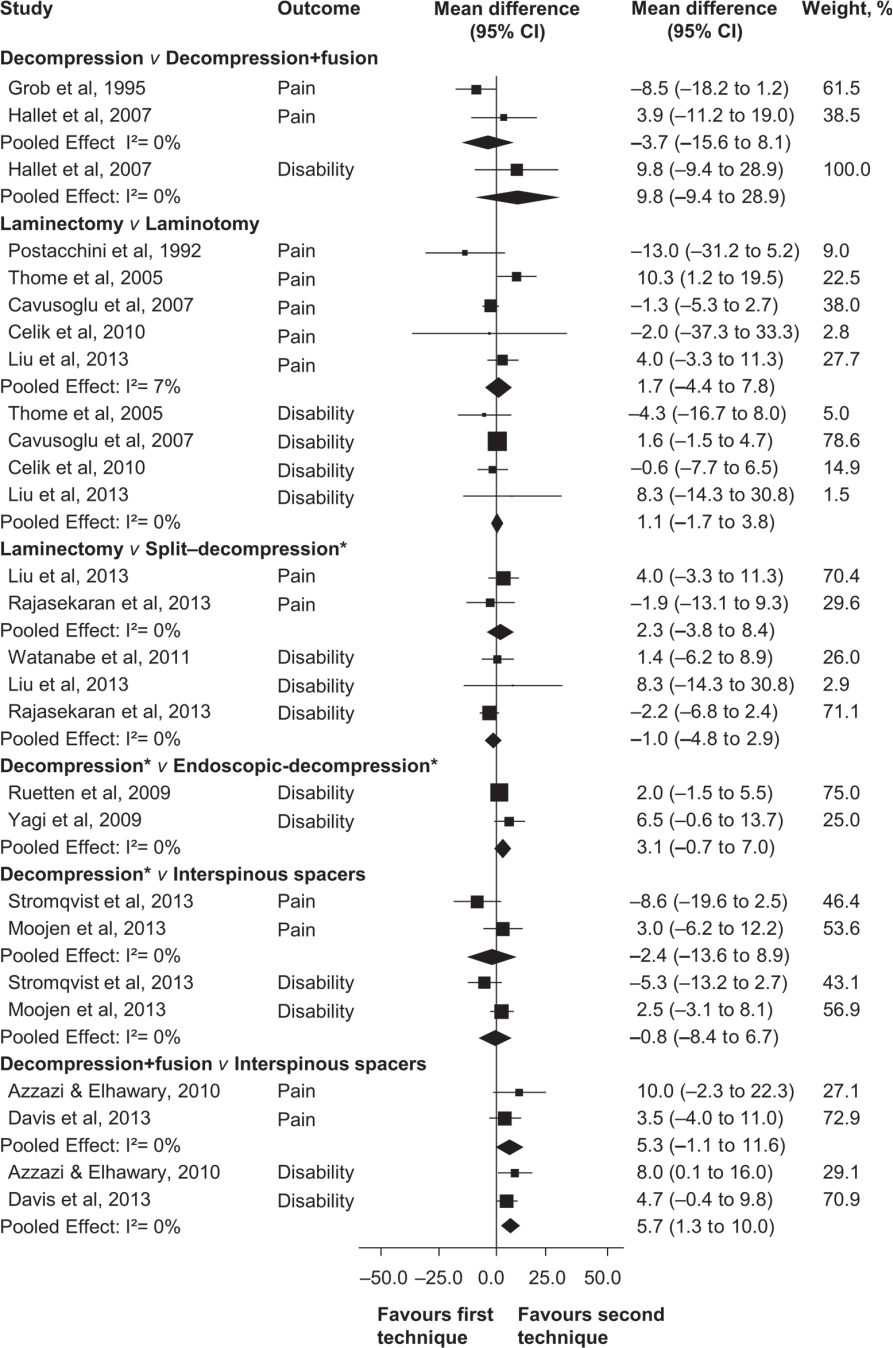
Mean Difference for Pain and Disability at Long-term Follow-up (12 months or more). *Decompression technique is laminectomy or laminotomy

No trials comparing surgery to no treatment or placebo/sham were identified. Therefore, all included trials compared different types of surgical techniques for lumbar spinal stenosis. Quality of evidence assessment and summary of findings, as well as the results of perioperative surgical outcomes (operation time, blood loss, and hospitalisation) are shown in Table 2. Pooled effect sizes for pain and disability at both short and long-term follow-up are presented in Figs 3 and 4.

**Table 2:** Summary of Findings and Quality of Evidence Assessment (GRADE).

**Summary of Findings**				**Quality of Evidence Assessment (GRADE)**			
**Outcomes**	**Time Point**	**No. Patients**	**Effect Size** ^a^ **(95% CI)**	**Study limitation**	**Consistency**	**Precision**	**Quality**
**Decompression *v* Decompression+Fusion**							
Pain	Long-term	86[9, 45]	–3.7 (–15.6 to 8.1)	Limitation (–1)	Inconsistency (–1)	Imprecision (–1)	Very low
Disability	Long-term	41[45]	9.8 (–9.4 to 28.9)	No limitation	One study (–1)	One study (–1)	Low
Walking ability^b^	Long-term	88[9, 44]	RR: 0.9 (0.4 to 1.9)	Limitation (–1)	Inconsistency (–1)	Imprecision (–1)	Very low
Operation time (min)	Perioperative	89[9, 45]	–105.2 (–227.6 to 17.3)	Limitation (–1)	No inconsistency	Imprecision (–1)	Low
Blood loss (mL)	Perioperative	89[9, 45]	**–826.5 (–1582.7 to—70.2)**	Limitation (–1)	Inconsistency (–1)	Imprecision (–1)	Very low
**Laminectomy *v* Laminotomy**							
Pain	Short-term	281[10, 36, 37]	–0.4 (–4.3 to 3.5)	No limitation	Inconsistency (–1)	Imprecision (–1)	Low
Pain	Long-term	393[10, 35–37, 39]	1.7 (–4.4 to 7.8)	Limitation (–1)	Inconsistency (–1)	No imprecision	Low
Disability	Short-term	333[10, 36–38]	1.6 (–1.0 to 4.2)	No limitation	No inconsistency	No imprecision	High
Disability	Long-term	335[10, 36, 37, 39]	1.1 (–1.7 to 3.8)	No limitation	Inconsistency (–1)	No imprecision	Moderate
Walking ability (m)	Short-term	233[36–38]	–7.6 (–37.4 to 22.3)	Limitation (–1)	Inconsistency (–1)	Imprecision (–1)	Very low
Walking ability (m)	Long-term	181[36, 37]	–3.0 (–32.7 to 26.7)	No limitation	No inconsistency	Imprecision (–1)	Moderate
Operation time (min)	Perioperative	279[35–37, 39]	–3.6 (–30.0 to 22.9)	Limitation (–1)	No inconsistency	Imprecision (–1)	Low
Blood loss (mL)	Perioperative	302[35–37, 39]	**34.1 (15.1 to 53.0)**	Limitation (–1)	No inconsistency	No imprecision	Moderate
**Laminectomy *v* Split–laminectomy/laminotomy**							
Pain	Long-term	105[39, 41]	2.3 (–3.8 to 8.4)	Limitation (–1)	Inconsistency (–1)	Imprecision (–1)	Very low
Disability	Long-term	148[39–41]	–1.0 (–4.8 to 2.9)	Limitation (–1)	Inconsistency (–1)	Imprecision (–1)	Very low
Recovery	Long-term	137[39–41]	2.1 (–5.7 to 9.8)	Limitation (–1)	Inconsistency (–1)	Imprecision (–1)	Very low
Operation time (min)	Perioperative	146[39–41]	–2.8 (–19.2 to 13.5)	Limitation (–1)	Inconsistency (–1)	Imprecision (–1)	Very low
Blood loss (mL)	Perioperative	146[39–41]	**21.8 (16.4 to 27.2)**	Limitation (–1)	No inconsistency	Imprecision (–1)	Low
Hospitalisation (days)	Perioperative	51[41]	–0.1 (–0.6 to 0.4)	No limitation	One study (–1)	One study (–1)	Low
**Laminectomy/laminotomy *v* Endoscopic–laminectomy/laminotomy**							
Disability	Short-term	202[42, 43]	5.2 (–2.2 to 12.5)	Limitation (–1)	No inconsistency	Imprecision (–1)	Low
Disability	Long-term	202[42, 43]	3.1 (–0.7 to 7.0)	Limitation (–1)	No inconsistency	Imprecision (–1)	Low
Operation time (min)	Perioperative	233[42, 43]	3.5 (–17.6 to 24.6)	Limitation (–1)	Inconsistency (–1)	Imprecision (–1)	Very low
Blood loss (mL)	Perioperative	41[43]	**34.0 (30.4 to 37.6)**	Limitation (–1)	One study (–1)	One study (–1)	Very low
Hospitalisation (days)	Perioperative	41[43]	**8.6 (6.8 to 10.3)**	Limitation (–1)	One study (–1)	One study (–1)	Very low
**Laminectomy/laminotomy *v* Interspinous process spacer device**							
Pain	Short-term	247[11, 46]	–4.8 (–11.1 to 1.5)	No limitation	No inconsistency	Imprecision (–1)	Moderate
Pain	Long-term	247[11, 46]	–2.4 (–13.6 to 8.9)	No limitation	Inconsistency (–1)	Imprecision (–1)	Low
Disability	Short-term	248[11, 46]	–0.4 (–6.9 to 6.2)	No limitation	Inconsistency (–1)	Imprecision (–1)	Low
Disability	Long-term	246[11, 46]	–0.8 (–8.4 to 6.7)	No limitation	Inconsistency (–1)	Imprecision (–1)	Low
Walking ability^b^	Short-term	145[46]	OR: 0.8 (0.4 to 1.3)	No limitation	One study (–1)	One study (–1)	Low
Walking ability^b^	Long-term	136[46]	OR: 1.3 (0.9 to 1.8)	No limitation	One study (–1)	One study (–1)	Low
Operation time (min)	Perioperative	259[11, 46]	**27.4 (10.8 to 44.1)**	No limitation	Inconsistency (–1)	Imprecision (–1)	Low
Hospitalisation (days)	Perioperative	159[46]	0.1 (–0.3 to 0.4)	No limitation	One study (–1)	One study (–1)	Low
**Decompression+Fusion *v* Interspinous process spacer device**							
Pain	Long-term	308[47, 49]	5.3 (–1.1 to 11.6)	Limitation (–1)	No inconsistency	No imprecision	Moderate
Disability	Long-term	308[47, 49]	**5.7 (1.3 to 10.0)**	Limitation (–1)	No inconsistency	No imprecision	Moderate
Operation time (min)	Perioperative	381[47, 49]	**78.8 (30.1 to 127.6)**	Limitation (–1)	No inconsistency	No imprecision	Moderate
Blood loss (mL)	Perioperative	320[49]	**238.9 (194.8 to 283.0)**	No limitation	One study (–1)	One study (–1)	Low
Hospitalisation (days)	Perioperative	382[47, 49]	**1.6 (0.9 to 2.3)**	Limitation (–1)	No inconsistency	No imprecision	Moderate

RR = risk ratio; OR = odds ratio; CI = confidence interval; m = metres; mL = millilitres; min = minutes.

^a^Effect size is mean difference, unless otherwise specified. Negative value favours first comparator. Effect sizes (95% CI) in bold indicate statistically significant results.

^b^Dichotomous data: Walking ability better (ability to walk 50% farther or increase of 80 m in the walking distance postoperatively) or same/worse, effect size reported as risk ratio or odds ratio.

### Decompression *v* Decompression plus fusion

The addition of fusion to bony decompression was investigated in three randomised trials reporting data from 133 patients at long-term follow-up [9, 44, 45]. Pooled analysis showed “very low quality” evidence of nonsignificant difference between treatment groups on pain reduction (MD—3.7, 95% CI—15.6 to 8.1). One trial revealed “low quality” evidence of no between-group difference for disability (MD 9.8, 95% CI—9.4 to 28.9). Two trials evaluated the effectiveness of decompression plus fusion compared to decompression alone on walking ability (i.e., patients were considered improved when able to increase their walking distance by 50% at follow-up). The analysis provided “very low quality” evidence of no difference on walking ability between groups (RR 0.9, 95% CI 0.4 to 1.9). Mean direct surgery costs was higher for patients treated by decompression plus fusion (USD $16,115) compared to decompression alone (USD $10,392). However, no inferential statistics were reported for this outcome.

### Laminectomy *v* Laminotomy

Six randomised controlled trials reporting data from 475 patients compared laminectomy to unilateral [10, 36, 38, 39], and bilateral laminotomies [35–37]. For pain, we found “low quality” evidence that laminotomy is not superior to laminectomy at short-term (MD—0.4, 95% CI—4.3 to 3.5) and long-term follow-up (MD 1.7, 95% CI—4.4 to 7.8). Likewise, “high” to “moderate quality” evidence revealed that laminotomy failed to show disability reduction when compared to laminectomy at short-term (MD 1.6, 95% CI—1.0 to 4.2) and long-term follow-up (MD 1.1, 95% CI—1.7 to 3.8). For short-term walking ability (i.e., walking distance in metres without radicular pain), there is “very low quality” evidence that laminotomy is not superior to laminectomy (MD—7.6, 95% CI—37.4 to 22.3), and “moderate quality” evidence of no difference at long-term follow-up (MD—3.0, 95% CI—32.7 to 26.7).

### Laminectomy *v* Split–laminectomy/laminotomy

Three trials reported data of 148 patients treated with bony decompression by laminectomy or with spinous process split–laminectomy/laminotomy at long-term follow-up [39–41]. Pooling showed no statistically significant difference between treatments for pain (MD 2.3, 95% CI—3.8 to 8.4) and disability (MD—1.0, 95% CI—4.8 to 2.9). We also found no difference on long-term recovery rate (MD 2.1, 95% CI—5.7 to 9.8) assessed by the Japanese Association Score (range, 0 to 100). The overall quality of evidence was rated as “very low quality” for all three outcomes, according to the GRADE criteria.

### Laminectomy/laminotomy *v* Endoscopic–laminectomy/laminotomy

The effectiveness of endoscopic–assisted laminectomy/laminotomy was investigated in two randomised trials including 233 patients [42, 43]. Pooling revealed “low quality” evidence of no significant effect of endoscopic approaches compared to conventional laminectomy/laminotomy on disability at short-term (MD 5.2, 95% CI—2.2 to 12.5), and long-term follow-up (MD 3.1, 95% CI—0.7 to 7.0). Pain intensity was not reported in these two studies.

### Laminectomy/laminotomy *v* Interspinous process spacer device

Two high methodological quality trials reported data of 259 patients comparing bony decompression by laminectomy or laminotomies to the X-Stop and Coflex interspinous process spacer devices [11, 46]. At short-term follow-up, “moderate quality” evidence showed no difference on pain reduction (MD—4.8, 95% CI—11.1 to 1.5). Likewise, “low quality” evidence revealed no long-term difference on pain between groups (MD—2.4, 95% CI—13.6 to 8.9). For disability, “low quality evidence” did not reveal any difference at short-term (MD—0.4, 95% CI—6.9 to 6.2) and long-term follow-up (MD—0.8, 95% CI—8.4 to 6.7). Additionally, one study showed “low quality” evidence of no benefit of interspinous spacers compared to decompression on walking ability (i.e., ability to walk 1200 m within 15 minutes or increase of 80 m compared to baseline walking distance) at short-term (OR 0.8, 95% CI 0.4 to 1.3) and long-term follow-up (OR 1.3, 95% CI 0.9 to 1.8).

### Decompression plus fusion *v* Interspinous process spacer device

Two trials compared decompression plus fusion to the X-Stop and Coflex devices [47, 49], including a total of 382 patients analysed at long-term follow-up only. There is “moderate quality” evidence of no difference between groups on pain reduction (MD 5.3, 95% CI—1.1 to 11.6). However, we found “moderate quality” evidence that interspinous spacers are slightly superior to decompression plus fusion on disability outcomes in the long-term (MD 5.7, 95% CI 1.3 to 10.0).

### Adverse events and reoperations

We found high variability in the number of reported adverse events across surgical techniques, with rates ranging from 4% to 45%. Trials reported a wide variety of minor and major surgical adverse events, ranging from transient urinary retention to cerebrovascular accident. Overall, reoperation rates ranged from 3% to 28%, with the interspinous process spacer devices revealing the highest rates.

“Very low” and “low quality” evidence revealed that patients undergoing decompression plus fusion had an overall higher rate of adverse events (20/64, 31% *v* 3/24, 13%; P = 0.07) and reoperations (9/92, 10% *v* 1/37, 3%; P = 0.47) when compared to decompression alone. This difference was not statistically significant, however. “Moderate quality” evidence showed that laminectomy had nonsignificant higher adverse events (23/154, 15% *v* 19/192, 10%; P = 0.60) and reoperations rates (6/68, 9% *v* 4/114, 4%; P = 0.12) than the minimally invasive laminotomies. We found “low” and “very low quality” evidence that conventional laminectomy revealed nonsignificant higher adverse event (3/23, 13% *v* 1/28, 4%, P = 0.25) and reoperation rates (1/38, 3% *v* 1/45, 2%, P = 0.90) than the spinous process splitting techniques. There is “very low quality” evidence that laminectomy/laminotomy result in significantly higher adverse event rates (16/100, 16% *v* 5/92, 5%; P = 0.03) than the endoscopic techniques. However, no difference in reoperation rates was observed (2/80, 3% *v* 3/81, 4%; P = 0.66). In trials investigating the effectiveness of interspinous process spacer devices, we found “moderate quality” evidence that bony decompression is not associated with higher adverse events (9/129, 7% *v* 6/130, 5%; P = 0.74). However, interspinous process spacers revealed a significantly higher reoperation rate (34/123, 28% *v* 9/122, 7%; P < 0.001). Trials comparing decompression plus fusion to interspinous process spacer devices reported similar rates of adverse events for both techniques (45%), and “low quality” evidence revealed no difference in rates of revision surgery (23/215, 11% *v* 8/107, 7%; P = 0.36).

## Discussion

The results of this systematic review have revealed a paucity of evidence on the efficacy of surgery for lumbar spinal stenosis, to date there are no published randomised controlled trials comparing surgery to no treatment or placebo/sham surgery. Placebo-controlled trials in surgery are feasible and powerful to show the efficacy of surgical procedures [51]. Therefore, we identified 17 published randomised trials that reported the comparative effectiveness of different surgical techniques. Our results show that overall there is no difference in the effectiveness among the most commonly used surgical techniques for lumbar spinal stenosis. More importantly, we have demonstrated that the addition of fusion to traditional decompression for the treatment of lumbar spinal stenosis adds no benefit in terms of pain or disability. We found that the interspinous process spacer devices showed better outcomes (disability, operation time, blood loss, and hospitalisation) compared to decompression plus fusion. However, interspinous spacers have significantly higher reoperation rates than bony decompression.

There are several strengths to our review. We have used a prespecified registered protocol, performed a sensitive electronic search on seven different databases, and selected studies with no restrictions for language or publication date. To our knowledge, this is the first review to objectively estimate the effectiveness amongst all surgical techniques for lumbar spinal stenosis focusing on patient-related outcomes, whereas past reviews performed pooled analysis based on surgeon-related outcomes (i.e., the effectiveness of a surgical technique was rated by the surgeon) [16]. Our review included only randomised clinical trials, as causal inference of treatment on clinical outcomes can only be made when patients are truly randomised to treatment groups [52]. A further limitation of past reviews is that many have drawn conclusions based on non-randomised trials (i.e., indirect comparisons, observational studies and case series) [53–55]. Although it is debatable whether meta-analysis from randomised trials can provide accurate estimates about harms of medical interventions [56, 57], this is the first review to assess the safety of all surgical techniques for lumbar spinal stenosis by investigating reported adverse events, reoperation rates, perioperative blood loss, operation time, and length of hospitalisation.

Our review has identified important weaknesses in the literature. Overall, the methodological quality of included studies was poor. Whereas blinding of the caregiver in surgical trials is typically not possible, only six trials reported blinding of outcome assessors and three studies reported that patients were blinded. The reporting of data was also poor among some included studies, and we had to estimate the treatment effect from graphs or by adopting data (e.g., standard deviation) from similar studies. We recommend that future trials follow the CONSORT statement when reporting randomised controlled trials [58]. The safety of surgical interventions also varied largely across studies and not all trials have reported the numbers of adverse events or reoperations. Therefore, it is possible we have underestimated the rates of complications and reoperations and alert that our conclusions on harms of included interventions should be interpreted with caution. Future studies should be more thorough in reporting these surgical outcomes [59]. Another limitation of our study is the inclusion of few studies in each meta-analysis and the variability of techniques used by surgeons.

We found no trials investigating the efficacy of surgery for lumbar spinal stenosis compared to placebo/sham surgery. Therefore its true efficacy rather than the effect of the patient's expectation of the surgical intervention (placebo effect) remains unknown. Given the amount of surgical techniques for the treatment of lumbar spinal stenosis the need for placebo/sham-controlled trials has never been greater. Previous work has proposed the appropriate ethical considerations for sham surgery [60], and demonstrated that placebo/sham-controlled trials in surgery are feasible [51]. For instance, sham-controlled trials have been recently published in investigating the efficacy of vertebroplasty for painful osteoporotic vertebral fractures [61]. In these trials, sham surgery was performed by inserting a blunt stylet and gently tapping the vertebral body. Likewise, Flum has suggested performing minimally invasive approaches to the spine, simulating the decompressive technique, but without actually removing any bone tissue [62]. The addition of fusion to decompression for spinal stenosis has been previously investigated in systematic reviews with conflicting conclusions [63, 64]. We have identified three randomised trials comparing decompression alone to decompression plus fusion, and our results revealed no significant differences between treatment groups on clinical outcomes. In fact, decompression plus fusion revealed significantly higher intraoperative blood loss when compared to decompression alone. These findings are based on “low” to “very low” quality evidence, however. One high quality trial revealed a cost difference of approximately USD $6,290 per patient for an additional fusion implant [45]. Therefore, the superiority of decompression plus fusion to decompression alone is still uncertain and surgeons should choose between these techniques with caution, especially considering the associated costs and perioperative complications of fusion. A systematic review has also investigated the effectiveness of interspinous process spacer devices for spinal stenosis, suggesting that spacer devices are superior to bony decompression [54]. However, this result was based on indirect comparisons through a network meta-analysis. Similarly, a second systematic review has failed to identify trials directly comparing these two techniques [53]. More recently, Wu et al reported results from meta-analyses that included both randomised and non-randomised studies [65]. In our review, pooling of two high methodological quality randomised trials has revealed no difference between treatments on pain, disability, or walking ability. Although the spacer devices showed significantly less operation time, they resulted in higher numbers of revision surgeries. Therefore, due to lack of effectiveness and higher reoperation rates of interspinous process devices compared to bony decompression, the recommendation for the use of decompressive devices is debatable.

## Conclusions

In conclusion, there is relatively limited evidence to guide the use of surgery for the management of lumbar spinal stenosis. Overall, the quality of the available evidence ranged from “high” to “very low” revealing nonsignificant differences across surgical techniques for lumbar spinal stenosis, and a small, but clinically debatable, benefit of interspinous spacer devices compared to decompression plus fusion. The addition of fusion to decompression is more costly, leads to more intraoperative blood loss, and fails to promote superior outcomes if compared to decompression alone. Although the operation using interspinous spacers is quicker, these devices are more expensive than conventional bony decompression and are associated with higher revision surgeries. We, therefore, question the use of decompression plus fusion and the safety of interspinous spacers in the management of patients with lumbar spinal stenosis. More high quality trials comparing the effectiveness between techniques are needed to support our findings. Patients and clinicians could use this review as an evidence-based tool to help decide the best surgical option for this condition.

## Supporting Information

S1 Checklist PRISMA Checklist. (DOC)

S1 Table Search Strategy. (DOCX)

S2 Table Risk of Bias (PEDro) of Included Studies. PEDro = Physiotherapy Evidence Database.
(DOCX)
