# Supplementary material for: Effectiveness of Surgery for Lumbar Spinal Stenosis: A Systematic Review and Meta-Analysis
Source: PLoS One. 2015 Mar 30;10(3):e0122800. doi: 10.1371/journal.pone.0122800 (PMC4378944; doi:10.1371/journal.pone.0122800)
Supplement: S1 Table — (DOCX) [file pone.0122800.s002.docx]

**Table S1. Search Strategy.**

| **MEDLINE, AMED and Cochrane Central Register of Controlled Trials (up to November 2014)** |
| --- |
| 1. (spinal stenosis or canal stenosis or (spin* adj3 stenosis) or (lumbar adj3 stenosis) or (lateral adj3 stenosis) or (central adj3 stenosis) or (foramin* adj3 stenosis) or neurogenic claudication or radiculopathy or radicular pain or lumbar radicular pain or spondylolisthesis or (lumb* adj5 spondyl*) or spondylosis).mp. [mp=title, subject heading word, registry word, abstract, trade name/generic name] 2. (general surgery or surgery or decompression or decompres* surgery or (spin* adj3 decompress*) or laminectomy or laminectom* or laminotom* or laminoplasty or spinal fusion or (spin* adj3 fusion) or (pedicle adj3 screw) or lumbar fusion or vertebrae fusion or vertebral fixation or spinal fixation or spondylodesis or spondylosyndesis or arthrodesis or arthrodesis or (posterolateral adj3 fusion) or (interbody adj3 fusion) or (anterior adj3 fusion) or (posterior adj3 fusion) or (transforaminal adj3 fusion) or (transpsoas adj3 fusion) or (facet adj3 fusion) or (bone adj3 graft) or (fixation adj3 spin*) or (pedicle adj3 fusion) or graft or (cage adj3 fusion) or (screw adj3 fusion) or foraminotomy or foraminectomy or surgical procedures, minimally invasive or minim* invasive).mp. [mp=title, subject heading word, registry word, abstract, trade name/generic name] 3. 1 and 2 4. (randomized controlled trial or (random* adj3 trial) or controlled clinical trial or randomized or placebo or randomly or random* or trial or clinical trial or clinical study).mp. [mp=title, subject heading word, registry word, abstract, trade name/generic name] 5. 3 and 4 6. Limit 5 to humans |
| **EMBASE (up to November 2014)** |
| 1. 'vertebral canal stenosis'/exp OR 'vertebral canal stenosis' OR 'spine NEAR/3 stenosis' OR 'lumbar NEAR/3 stenosis' OR 'lateral NEAR/3 stenosis' OR 'central stenosis' OR 'foraminal stenosis' OR 'neurogenic claudication' OR 'radiculopathy'/exp OR radiculopathy OR 'radicular pain'/exp OR 'radicular pain' OR 'lumbar radicular pain' OR 'spondylolisthesis'/exp OR spondylolisthesis OR 'spondylosis'/exp OR spondylosis 2. 'surgery'/exp OR surgery OR 'decompression surgery'/exp OR 'decompression surgery' OR 'decompression spinal cord'/exp OR 'decompression spinal cord' OR 'decompression'/exp OR decompression OR 'laminectomy'/exp OR laminectomy OR laminotomy OR 'laminoplasty'/exp OR laminoplasty OR 'spine fusion'/exp OR 'spine fusion' OR 'spinal fusion'/exp OR 'spinal fusion' OR 'lumbar NEAR/3 fusion' OR 'vertebrae fusion' OR 'vertebral fixation' OR 'spondylodesis'/exp OR spondylodesis OR 'spinal fixation' OR 'spinal fixation device'/exp OR 'spinal fixation device' OR 'spondylosyndesis'/exp OR spondylosyndesis OR posterolateral NEAR/3 fusion OR interbody NEAR/3 fusion OR anterior NEAR/3 fusion OR posterior NEAR/3 fusion OR transforaminal NEAR/3 fusion OR 'transpsoas fusion' OR facet NEAR/3 fusion OR 'arthrodesis'/exp OR arthrodesis OR bone NEAR/5 graft OR fixation NEAR/5 spin* OR pedicle NEAR/5 fusion OR cage NEAR/5 fusion OR screw NEAR/5 fusion OR pedicle NEAR/5 screw OR 'foraminotomy'/exp OR foraminotomy OR foraminectomy OR 'minimally invasive procedures'/exp OR 'minimally invasive procedures' OR 'minim$ invasive' 3. #1 AND #2 4. 'randomized controlled trial'/exp OR 'randomized controlled trial' OR 'controlled clinical trial'/exp OR 'controlled clinical trial' OR 'clinical trial'/exp OR 'clinical trial' OR randomized:ab OR placebo:ab OR randomly:ab OR trial:ab OR 'clinical study':ab 5. #3 AND #4 6. 5 AND 'human'/de |
| **CINAHL (up to November 2014)** |
| 1. (MH "Spinal Stenosis") or "spinal stenosis" or "spin* stenosis" or "canal stenosis" or "lumbar stenosis" or "lateral stenosis" or "central stenosis" or "foramin* stenosis" or (MH "Intermittent Claudication") or "neurogenic claudication" or (MH "Radiculopathy") or "Radiculopathy" or "radicular pain" or "lumbar radicular pain" or (MH "Spondylolisthesis") or "Spondylolisthesis" or "lumb* spondyl*" or (MH "Spondylosis+") or "Spondylosis" or (MH "Spondylolysis+") or "spondilolisys" 2. (MH "Surgery, Operative+") or "surgery" or (MH "Decompression, Surgical+") or "Decompression" "Decompres* surgery" or "spin* decompress*" or "lumbar decompress*" or (MH "Laminectomy") or "Laminectomy" or "Laminectom*" or "Laminotom*" or "Laminoplasty" or (MH "Spinal Fusion") or "Spinal Fusion" or (MH "Arthrodesis+") or "arthrodesis" or "spin* fusion" or (MH "Orthopedic Fixation Devices+") or "pedicle screw" or "lumbar fusion" or "vertebrae fusion" or "vertebral fixation" or "Spondylodesis" or "Spinal fixation" or (MH "Orthopedic Fixation Devices+") or "Spondylosyndesis" or "Posterolateral fusion" or "Interbody fusion" or "Anterior near/5 fusion" or "Anterior fusion" or "Posterior fusion" or "Transforaminal fusion" or "Transpsoas fusion" or "Facet fusion" or (MH "Arthrodesis+") or (MH "Grafts+") OR "Bone graft" or "Pedicle fusion" or "Cage fusion" or "Screw fusion" or "Foraminotomy" or "Foraminectomy" or (MH "Minimally Invasive Procedures") or "Minimally Invasive" 3. 1 and 2 |
| **Web of Science (up to November 2014)** |
| 1. "spin* stenosis" or "canal stenosis" or "lumbar stenosis" or "lateral stenosis" or "central stenosis" or "foramin* stenosis" or "neurogenic claudication" or "radiculopathy" or " radicular pain" or "lumbar radicular pain" or "spondylolisthesis" or Spondylosis or Spondylolysis 2. Surgery or Decompression or Decompressive or Laminectomy or Laminotom* or Laminoplasty or "spin* fusion" or "lumbar fusion" or Arthrodesis or "vertebrae fusion" or "vertebral fixation" or "spinal fixation" or "posterolateral fusion" or "interbody fusion" or "anterior fusion" or "posterior fusion" or "transforaminal fusion" or "transpsoas fusion" or "facet fusion" or Arthrodesis or "cage fusion" or "pedicle screw" or Foraminotomy or Foraminectomy or "minimally invasive" 3. 1 and 2 4. "randomized controlled trial" or "randomized clinical trial" or "controlled clinical trial" or Randomized or Placebo or Random* or Trial or "clinical trial" or "clinical study" 5. 3 and 4 |
| **LILACS (up to November 2014)** |
| ("spine stenosis" OR "spinal stenosis" OR "canal stenosis" OR "lumbar stenosis" OR "central stenosis" OR "lateral stenosis" OR "foraminal stenosis" OR "spondylolisthesis" OR spondylosis OR "neurogenic claudication" OR radiculopathy OR "radicular pain") AND (surgery OR decompression OR decompressive OR laminectomy OR laminotomy OR laminoplasty OR "spinal fusion" OR "spine fusion" OR arthrodesis OR "lumbar fusion" OR "vertebrae fusion" OR "vertebral fixation" OR spondylodesis OR "spinal fixation" OR spondylosyndesis OR "posterolateral fusion" OR "interbody fusion" OR "anterior fusion" OR "posterior fusion" OR "transforaminal fusion" OR "transpsoas fusion" OR "facet fusion" OR "bone graft" OR "pedicle fusion" OR "cage fusion" OR "screw fusion" OR "pedicle screw" OR screw OR rod OR foraminotomy OR foraminectomy OR "surgical procedure" OR "minimally invasive"). |
