# Supplementary material for: Effectiveness of Surgery for Lumbar Spinal Stenosis: A Systematic Review and Meta-Analysis
Source: PLoS One. 2015 Mar 30;10(3):e0122800. doi: 10.1371/journal.pone.0122800 (PMC4378944; doi:10.1371/journal.pone.0122800)
Supplement: S2 Table — PEDro = Physiotherapy Evidence Database. (DOCX) [file pone.0122800.s003.docx]

**Table S2. Risk of Bias (PEDro) of Included Studies.**

| **Study** | **Item 1** | **Item 2** | **Item 3** | **Item 4** | **Item 5** | **Item 6** | **Item 7** | **Item 8** | **Item 9** | **Item 10** | **Total** |
| --- | --- | --- | --- | --- | --- | --- | --- | --- | --- | --- | --- |
| Postacchini et al, 1992 | Yes | No | No | No | No | Yes | Yes | No | Yes | No | 4 |
| Thome et al, 2005 | Yes | Yes | Yes | No | No | No | Yes | No | Yes | Yes | 6 |
| Cavusoglu et al, 2007 | Yes | Yes | Yes | Yes | No | Yes | Yes | No | Yes | Yes | 8 |
| Celik et al, 2010 | Yes | Yes | Yes | No | No | No | Yes | No | Yes | Yes | 6 |
| Gurelik et al, 2012 | Yes | No | No | No | No | No | Yes | No | Yes | Yes | 4 |
| Liu et al, 2013 | Yes | No | Yes | No | No | No | Yes | No | Yes | Yes | 5 |
| Watanabe et al, 2011 | Yes | No | Yes | No | No | No | No | No | Yes | Yes | 4 |
| Rajasekaran et al, 2013 | Yes | Yes | Yes | No | No | Yes | Yes | No | Yes | No | 6 |
| Ruetten et al, 2009 | Yes | Yes | Yes | No | No | No | Yes | No | Yes | No | 5 |
| Yagi et al, 2009 | Yes | No | Yes | No | No | No | No | No | Yes | Yes | 4 |
| Bridwell et al, 1993 | Yes | No | No | No | No | No | Yes | No | Yes | No | 3 |
| Grob et al, 1995 | Yes | No | No | No | No | Yes | Yes | No | Yes | No | 4 |
| Hallet et al, 2007 | Yes | Yes | Yes | No | No | Yes | Yes | Yes | Yes | Yes | 8 |
| Stromqvist et al, 2013 | Yes | Yes | Yes | No | No | No | Yes | Yes | Yes | Yes | 7 |
| Moojen et al, 2013 | Yes | Yes | Yes | Yes | No | Yes | Yes | Yes | Yes | Yes | 9 |
| Azzazi et al, 2010 | Yes | No | Yes | No | No | No | Yes | No | Yes | No | 4 |
| Davis et al, 2013 | Yes | Yes | Yes | Yes | No | No | Yes | No | Yes | Yes | 7 |

PEDro = Physiotherapy Evidence Database; Item 1 = random allocation; Item 2 = concealed allocation; Item 3 = baseline comparability; Item 4 = blinding of subjects; Item 5 = blinding of therapists; Item 6 = blinding of assessors; Item 7 = adequate follow-up; Item 8 = intention-to-treat analysis; Item 9 = between-group comparisons; Item 10 = point estimates and variability.
